# Targeting NMDA receptor in Alzheimer’s disease: identifying novel inhibitors using computational approaches

**DOI:** 10.3389/fphar.2023.1208968

**Published:** 2023-06-21

**Authors:** Arif Jamal Siddiqui, Riadh Badraoui, Sadaf Jahan, Mohammed Merae Alshahrani, Maqsood Ahmed Siddiqui, Andleeb Khan, Mohd Adnan

**Affiliations:** ^1^ Department of Biology, College of Science, University of Hail, Hail, Saudi Arabia; ^2^ Department of Medical Laboratory Sciences, College of Applied Medical Sciences, Majmaah University, Al Majmaah, Saudi Arabia; ^3^ Department of Clinical Laboratory Sciences, Faculty of Applied Medical Sciences, Najran University, Najran, Saudi Arabia; ^4^ Department of Zoology, College of Science, King Saud University, Riyadh, Saudi Arabia; ^5^ Department of Pharmacology and Toxicology, College of Pharmacy, Jazan University, Jazan, Saudi Arabia

**Keywords:** NMDA receptor, Alzheimer’s, computer biology, dynamics, pharmacophore, free energy calculation

## Abstract

The glutamate-gated ion channels known as N-methyl-d-aspartate receptors (NMDARs) are important for both normal and pathological brain function. Subunit-selective antagonists have high therapeutic promise since many pathological conditions involve NMDAR over activation, although few clinical successes have been reported. Allosteric inhibitors of GluN2B-containing receptors are among the most potential NMDAR targeting drugs. Since the discovery of ifenprodil, a variety of GluN2B-selective compounds have been discovered, each with remarkably unique structural motifs. These results expand the allosteric and pharmacolog-ical spectrum of NMDARs and provide a new structural basis for the development of next-generation GluN2B antagonists that have therapeutic potential in brain diseases. Small molecule therapeutic inhibitors targeting NMDA have recently been developed to target CNS disorders such as Alzheimer’s disease. In the current study, a cheminformatics method was used to discover potential antagonists and to identify the structural requirements for Gly/NMDA antagonism. In this case we have created a useful pharmacophore model with solid statistical values. Through pharmacophore mapping, the verified model was used to filter out virtual matches from the ZINC database. Assessing receptor-ligand binding mechanisms and affinities used molecular docking. To find the best hits, the GlideScore and the interaction of molecules with important amino acids were considered essential features. We found some molecular inhibitors, namely, ZINC13729211, ZINC07430424, ZINC08614951, ZINC60927204, ZINC12447511, and ZINC18889258 with high binding affinity using computational methods. The molecules in our studies showed characteristics such as good stability, hydrogen bonding and higher binding affinities in the solvation-based assessment method than ifenprodil with acceptable ADMET profile. Moreover, these six leads have been proposed as potential new perspectives for exploring potent Gly/NMDA receptor antagonists. In addition, it can be tested in the laboratory for potential therapeutic strategies for both *in vitro* and *in vivo* research.

## 1 Introduction

Synaptic transmission is important for the nervous system’s ability to process and store information. Synapses are particular junctions between neurons where presynaptic neurons induce postsynaptic neurons to express neurotransmitter receptors. The amino acid glutamate plays an important role in mediating excitatory synaptic neurotransmission by involving two distinct glutamate receptor subtypes: ionotropic and metabotropic. Ion channels controlled by ligands are known as ionotropic glutamate receptors (Hansen and Traynelis, 2011; Swanger et al, 2018; Swanger and Traynelis, 2018; Benke and Traynelis, 2019). The N-methyl-D-aspartate (NMDA) receptors are excitatory neurotransmitter-activated, ligand-gated ion channels. Because they are mainly found at excitatory synapses, these N-methyl-D-aspartate receptors (NMDAR) play a role in excitatory neurotransmission in the central nervous system. Besides generating an electrical signal, open NMDARs also induce calcium influx, which is essential for synaptic signaling and neuroplasticity in learning and memory (Wang and Reddy, 2017; Swanger et al, 2018; Benke and Traynelis, 2019). However, activation of extrasynaptic NMDARs causes excitotoxicity and promotes cell death, underlying a possible mechanism of neurodegeneration occurring in Alzheimer’s disease (AD) (Thal et al, 2015; Lopez and Kuller, 2019; Lei et al, 2021). Abnormalities in both structure and function are part of the pathogenesis of AD. As AD worsens, the brain develops numerous anatomical abnormalities, such as senile plaques of A and neurofibrillary tangles of phosphorylated tau, and significant loss of synaptic profiles (Perl, 2010). To date, AD has no known treatment. However, there are some therapies. The National Institute on Aging’s Alzheimer’s disease Medications Fact Sheet lists two groups of FDA-approved prescription drugs currently used to treat AD patients. For mild to severe AD, cholinesterase inhibitors are an option. The other contains memantine, an antagonist to NMDARs, a receptor controlled by the neurotransmitter glutamate, and is used to treat moderate to severe Alzheimer’s disease. Neuronal cell viability is compromised by inadequate synaptic NMDAR signaling. However, excessive glutamatergic signaling stimulation causes excitotoxicity, in which nerve cells are damaged or destroyed, or neurological damage such as stroke (Rothman and Olney, 1986). Numerous studies indicate that glutamate excitotoxicity plays a role in delayed, steadily progressing neurodegeneration in addition to the acute effect (Lipton and Rosenberg, 1994; Chifor et al, 2022). Because NMDARs have much higher calcium ion permeability than other iGluRs, increasing evidence suggests that toxicity is mainly mediated by excessive Ca2+ entry, mainly via NMDARs (Daw et al, 1993; Liu et al, 2004; Myoga and Regehr, 2011; Tan et al, 2022). In this regard, the slight depolarization of the postsynaptic membrane, as well as other factors that unblock Mg2+, can mildly and chronically activate NMDARs, leading to prolonged Ca2+ influx into the postsynaptic neuron. This in turn justifies clinical testing of memantine, an NMDAR antagonist, for the symptomatic and protective treatment of AD. Pathologic Ca2+ signaling leads to a gradual loss of synaptic function and eventual neuronal cell death, which clinically correlates with a gradual decline in cognition/memory and the establishment of pathologic neural anatomy seen in AD patients (Danysz et al, 1995; Day and Langston, 2006; Czapski and Strosznajder, 2021). Therefore, the amount of NMDAR signaling must be high since NMDARs are also crucial for cell survival. Some of the inhibitors for NMDAR have been reported in previous reviews (Danysz et al, 1995; Ates-Alagoz and Adejare, 2013; Davoudian and Wilkinson, 2020). The following side effects are possible with NMDA antagonists, including high blood pressure, confusion, headache, constipation, cough, back pain, pain, lightheadedness (daytime sleepiness), vomiting, yawning and dyspnea (shortness of breath), fatigue. There is still a lack of inhibitors for NMDA with high potency and low side effects. There has been some reports which have suggested the use of various computational approaches for identifying inhibitors (Sharma et al, 2020; Sharma et al, 2021; Sharma et al, 2022). Therefore, there is a need for new approaches to identify new inhibitors for NMDARs. Our current work here focuses on computational methods that open a new field for designing novel, highly potent inhibitors against the enzyme and predicting potential inhibitors of NMDAR using these structural details.

## 2 Materials and methods

### 2.1 Binding site analysis

The RCSB-PDB database (https://www.rcsb.org/) contains a number of molecules with their inhibitory activity and bound complexes with NMDAR (Burley et al, 2018). The bound conformation of this compound was used to understand the critical protein-ligand interaction. Through these studies, we were able to identify critical residues important for NMDAR inhibition, which could play a crucial role in the design and optimization of new inhibitors.

### 2.2 Pharmacophore model generation and validation

The RCSB-PDB database contains a number of molecules with their inhibitory effects and bound complexes with NMDARs. The bound conformation of this compound was used to generate structure-ligand based pharmacophore theory. Using the pharmacophore modeling based screening approach initially in our workflow, led us to shortlist hit molecules having 3D features similar to the already known inhibitor molecules in the initial step. Thus, the approach would help us to identify hit molecules having 3D based molecular features. Schrödinger’s PHASE module was used to develop the pharmacophore hypothesis (Dixon et al, 2006). It uses variable molecular orientation through superimposition and adjustment to similarity constraints. Spin angles of the dangling bonds are included in the conformational data to probe molecular alignments. The conformation of the molecule that satisfies the constraints is allowed to generate additional conformations that are similar. The similarity and quantity of aligned features, the volume of that alignment, and the van der Waals energy of the conformation are used to assess the suitability of the alignment. Distance and angle constraints are introduced and pharmacophoric features are extracted. To verify the pharmacophore theory, a test kit database was created consisting of these 20 recognized NMDA inhibitors added to 380 molecules derived from the DUDE database and treated as inactive (Mysinger et al, 2012). This test database was used to verify that a model could discriminate between active ingredients and baits. Calculated metrics such as enrichment factor (EF) and goodness of hit score (GH) are critical in determining the caliber of pharmacophore theory produced. A flexible search approach was implemented for screening purposes.

### 2.3 Virtual screening of ZINC database

The ZINC database was utilized for our simulated screening (Tingle et al, 2023). For virtual screening, the pharmacophoric model that had the most significant statistical parameters was selected. The resultant hit molecules which were screened using a structure-based pharmacophore model were subsequently added to a new database.

### 2.4 Molecular docking

The atomic structure of NMDA linked to the Ifenprodil ligand (PDB ID-5ewj) was used for docking studies (Stroebel et al, 2016). The docking tests were carried out with the docking program GLIDE (Friesner et al, 2004). Redocking experiments were performed first after the pharmacophore-based screen to optimize settings for an additional docking-based virtual screening of hit compounds. Docking experiments were conducted to find potential hit substances. This required preparing the protein, the ligand, creating the lattice and studying the docking pose.

### 2.5 Molecular dynamics simulation

The stability with time of the bound orientations of the chemicals in the receptor’s binding pocket can be assessed using molecular dynamics (MD). Its usefulness in locating new inhibitors has been demonstrated in numerous experiments (Sharma and Siddiqi, 2019; Siddiqui et al, 2021; Xiong et al, 2021; Sharma et al, 2022; Siddiqui et al, 2023a; Siddiqui et al, 2023b; Siddiqui et al, 2023c). The gromacs 2020 version was used for MD research (Sharma et al, 2021). MD experiments were performed using the CHARMM force field (Páll et al, 2020). The CHARMM parameters of the ligand were recorded using the SwissParam web service (Vanommeslaeghe and MacKerell Jr, 2015). Using the TIP3P water model and the CHARMM force field, we first create the structure of the protein. A cage was defined around the protein-ligand complex with a radius of 12 cubic. Using counterions, the solvated compound was neutralized. The energy consumption of the system was further reduced for 50,000 steps by the steepest descent technique. The system was then calibrated for 200 ps using NVT and NPT based on the Leap Frog algorithm. The system was exposed to an MD of 200 ns after equilibration. After MD, the system was scaled down by removing the periodic boundary conditions, and then examined for link stability and other investigations. UCSF chimera software was used for the MD study (Pettersen et al, 2004; Zoete et al, 2011). Plots were generated after analysis using the XMGRACE tool [https://plasma-gate.weizmann.ac.il/Grace/].

### 2.6 Solvent based binding energy calculations

The post-processing final state method known as MM-PBSA is used to determine the free energies of compounds in solution. Using ensembles created from MD or Monte Carlo simulations, Python software MMPBSA.py streamlines final state free energy calculations. With MMPBSA.py, a number of implicit solvation models are accessible, such as the Reference Interaction Site Model, the Generalized Born Model, and the Poisson-Boltzmann Model. To approximate the entropy of the solute, vibrational frequencies can be calculated using normal mode analysis or quasi-harmonic analysis. Specific interactions can also be unraveled using alanine scanning or free energy analysis. By evenly distributing frames across available processors, a parallel implementation significantly speeds up computation. Effective and easy to use, MMPBSA.py tool is flexible enough to meet the needs of users performing final state free energy calculations. Solvent-based binding analysis of the protein-ligand complexes was performed using the method described in various previous studies using the GMX MMPBSA tool (Valdés-Tresanco et al, 2021).

## 3 Results and discussion

### 3.1 Binding site analysis

**FIGURE 1:**
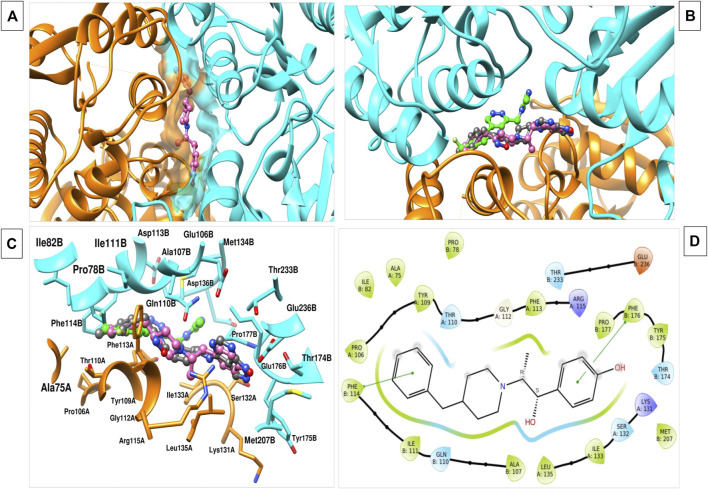
**(A)** The image indicates the binding interface of the two chains of NMDAR and ifenprodil. **(B)** Various inhibitors, namely, ifenprodil (hot pink), MK-22 (dark grey), EVT-101 (green) binding to the same interface with varied conformation. **(C)** 3d interaction plot of inhibitor with the residues at interface. **(D)** 2d interaction plot of the ifenprodil and various interacting residues within the 5 Å radius.

The RCSB-PDB database contains a number of molecules with their inhibitory activity and bound complexes with NMDAR, namely, 5EWJ, 5EWL, 5EWM (Stroebel et al, 2016). The bound conformation of this compound was used to understand the critical protein-ligand interaction (Figures 1A–D). Through these studies, we were able to identify critical residues important for NMDAR inhibition, which could play a crucial role in the design and optimization of new inhibitors. We observed that most of the reported antagonists for NMDAR bind to the cleavage site of two chains of NMDAR, namely, chain A (GLUN1) and chain B (GLUN2B). residues of chain A, namely, A75, P106, Y109, T110, G112, W113, R115, K131, S132, I133, L135 and residues of chain B, namely, P78, I82, A107, N110, I111, W114, T174, Y175, W176, P177, M207, T233, D236 play an important interaction role. These two B-chain residues Phe114, Phe176 were observed to form a pi-pi stacking interaction with the ifenprodil inhibitor. In general, molecules with hydrophobic moieties towards the two opposite sites showed a strong interaction pattern. The same was observed for the molecules MK-22 and EVT-101. Comparing the binding pocket of glutamate with NMDAR, it was observed that it is separate and can regulate NMDAR in different ways (Zhu et al, 2016; Yu and Lau, 2018). Therefore, it can be concluded that inhibitor molecules with the same molecular features similar to ifenprodil and related molecules that bind to the A-chain and B-chain interface can be used as antagonists to treat AD.

### 3.2 Pharmacophore model generation and validation

**FIGURE 2:**
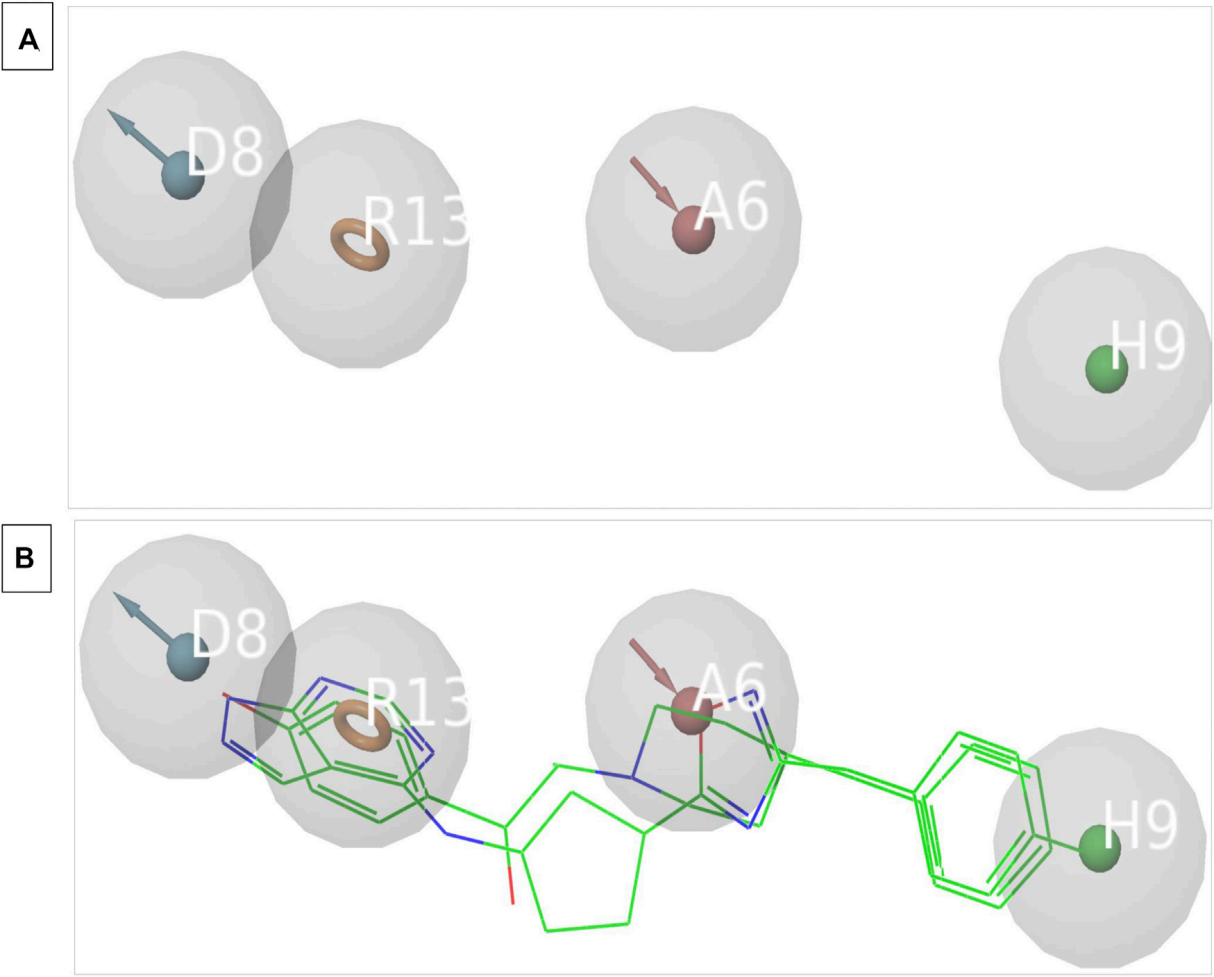
**(A)** The image indicates the 4 feature pharmacophore model. **(B)** The image indicates the overlapped structure of pharmacophore model and the known inhibitors of NMDAR which are bound to the interface.

3D structure determination of proteins is required for drug design and today the most established structure can be found in various protein databases or via homology modelling. The molecular structure of NMDAR in a linker complex (PDB: 5EWJ, 5EWL, 5EWM) was identified and a structural ligand-based pharmacophore model was constructed. Using an IC_50_ value and X-ray diffraction, the empirically determined affinity of the chosen ligand for NMDAR protein was confirmed. The inhibitor can control overall expression by binding to NMDAR. In some cases, poor potency of an inhibitor against a particular protein can be caused by unstable inhibitor binding. In order to identify the active series of inhibitors, they must interact appropriately to result in significantly higher biological activity compared to the current inhibitors. Using the Schrödinger PHASE module, the structural ligand-based pharmacophore models were used to generate important chemical properties. A variety of models were created and model 6 was selected for its highest score (Table 1). The different molecular properties were identified and a total of 7 were found. Among them, a protein-ligand complex interaction was shown that involved two hydrophobes, two aromatic ring bonds, 2 H-bond acceptors, one H-bond donor, and a few void volume features. Some features were omitted from the pharmacophore model to retain the best ligand-based pharmacophore features. By carefully analyzing the MD pattern of the top ligands, we observed that hydrophobic features and aromatic features can play crucial roles in the interaction. Therefore, more weight has been added to these features. Also, placing a donor group can increase the interaction of the ligand by interacting with the residue, namely, Glu236B. Also the acceptor group can increase the interaction by interacting with the Arg115A side chain. Other features have been mutated to become a top model. Our top model consisted of 4 traits including a hydrophobic trait (H9), an aromatic trait (R13), a donor trait (D8), and an acceptor trait (A6) (Figure 2). Verified pharmacophore analysis is required to ensure the accuracy of the pharmacophore analysis and to confirm its validity. Prior to database screening, a structure-based pharmacophore model developed in this research was tested for its ability to discriminate between drugs and mimics. The pharmacophore model was verified using 20 recognized NMDAR inhibitors. 380 baits were added from the DUDE database. The structural ligand-based model built above was the modified model 6 with a fidelity of 0.82. This was the highest score we could achieve with a structural ligand-based model. Different statistical parameters of different models are summarized in Table 1. For the top models we found that adding tolerance to the distance constraints decreases the accuracy of the pharmacophore model. Therefore, optimal distance constraints were used for top models.

**TABLE 1:** Statistical parameters for screening of NMDA test set molecules using pharmacophore models.

Parameters	Model-1	Model-2	Model-3	Model-6
Total no. of molecule s in database (D)	400	400	400	400
Total no. of active (A)	20	20	20	20
Total hits (H_t_)	43	48	31	24
Active hits (H_a_)	17	16	14	19
% yield of actives (H _a_/H _t_ × 100)	39	33.33	45.16	79.17
% ratio of actives (H _a_/A × 100)	85	80	70	95
Enrichment factor (EF)	7.90	6.67	9.03	15.83
False positives (H_t_ - H_a_)	26	32	17	5
False negatives (A- H_a_)	3	4	6	1
Goodness of hit score (GH)	0.47	0.26	0.49	0.82

### 3.3 Virtual screening of ZINC database

After virtual screening of the ZINC database using the above structure-ligand based pharmacophore model, only 38,105 hits were screened out, filtering out the remaining molecules. So, after the pharmacophore-based screen, we only had 38,105 molecules with pharmacophoric characteristics.

### 3.4 Molecular docking and ADMET analysis

The GLIDE docking suite was used to perform docking experiments using the atomic structure of Ifenprodil attached to NMDAR. To optimize the variables by removing the ligand ifenprodil from the active site, redocking experiments were initially performed. The structure of the compound was recreated using the Marvin sketch, and redocking experiments were performed using the 3D conformation of the compound. As we noticed, our re-docked pose had a slight deviation of 0.819 (Supplementary Figure S1). After performing redocking studies to optimize the docking study settings, we performed docking studies on all 38,105 molecules that passed the pharmacophore-based virtual screening using the HTVS scoring method. Also, we selected top molecules with higher binding affinity as the control molecule for XP-based docking, which is a more robust method to determine the binding positions of ligands with respect to protein. The table below summarizes the binding energies of the top 10 molecules and the control molecule (Table 2). According to careful conformational analysis, all hits with higher or similar binding energies than the ifenprodil blocker interacted with this binding pocket. (Figures 3.1–3.10). The majority of molecules interfere in some way with the hydrophobic cavity composed of A chain residues, namely, A75, P106, Y109, T110, G112, W113, R115, K131, S132, I133, L135, and B chain residues, namely, P78, I82, A107, N110, I111, W114, T174, Y175, W176, P177, M207, T233, D236. In most molecules, part of their unit interacts with the hydrophobic pocket composed of residues Ala75, I133, L135 of chain A and I111, W114, P177, and W176 of chain B. Some molecules showed hydrogen bonding interactions with the residues, mainly Tyr109 of chain A and Gln110, Asp206, Met207, Glu235, Glu236 of chain B with different units or parts of their total molecular structure. The other main interaction we found in most residues was the Pi-Pi, Pi-cation interaction in some of the ligands with the residues, namely, Phe114, Phe176 of chain B and Tyr109, Arg115 of chain A. All of it was found that the top 10 compounds have higher binding energies than ifenprodil, and they also showed interaction with residues that were found to interact with control ligands. Also, some new interaction especially hydrogen bond interaction with Glu236 residue of chain B and cation-Pi interactions with Arg115 of chain A were some of the new observed interactions. Furthermore, these compounds shared pharmacophoric properties with the active ingredients, which prompted us to conduct additional research on these molecules. All of the molecules selected above have an acceptable golden triangle rule like the control molecule Ifenprodil. The ADMET profile values ​​of the molecules are summarized in Table 2. MD studies, discussed in the next section, were performed to verify the stability of these top 10 chemicals in the binding pocket over time.

**TABLE 2:** The table summarizes the Glide score, ADMETvalues and GMX MMPBSA score of various top shortlisted hit molecules after docking based evaluation. GMX MMPBSA score was calculated using last 50 ns trajectory of the MD simulation file.

Sr No.	ZINC database code	Code used	Glide score	logP	F_30%_	BBB* Penetration	H-HT**	T 1/2***	Golden triangle	GMX MMPBSA (kcal/mol)
1	ZINC19211094	1	−14.46	2.432	0.003	0.92	0.939	0.148	Accepted	−24.6641
2	ZINC32960625	2	−13.29	4.482	0.037	0.046	0.555	0.361	Accepted	−28.5832
3	ZINC13729211	3	−13.1	2.688	0.14	0.631	0.869	0.31	Accepted	−42.4383
4	ZINC07430424	4	−12.88	3.336	0.82	0.118	0.099	0.896	Accepted	−36.2913
5	ZINC08614951	5	−12.67	4.012	0.007	0.106	0.41	0.413	Accepted	−38.9079
6	ZINC91665734	6	−12.58	1.046	0.957	0.423	0.774	0.683	Accepted	−32.7661
7	ZINC20877855	7	−12.57	4.333	0.02	0.098	0.324	0.555	Accepted	−32.9327
8	ZINC60927204	8	−12.56	2.212	0.157	0.16	0.446	0.92	Accepted	−36.8264
9	ZINC12447511	9	−12.55	3.163	0.036	0.748	0.975	0.484	Accepted	−40.7174
10	ZINC18889258	10	−12.52	5.18	0.061	0.01	0.09	0.133	Accepted	−40.8428
11	**Ifenprodil**	Control	−10.29	3.549	0.881	0.947	0.208	0.552	Accepted	−30.7786

* BBB, Blood Brain Barrier; ** H-HT, High Hepatotoxicity; *** T1/2, Half Excretion Time.

**FIGURE 3:**
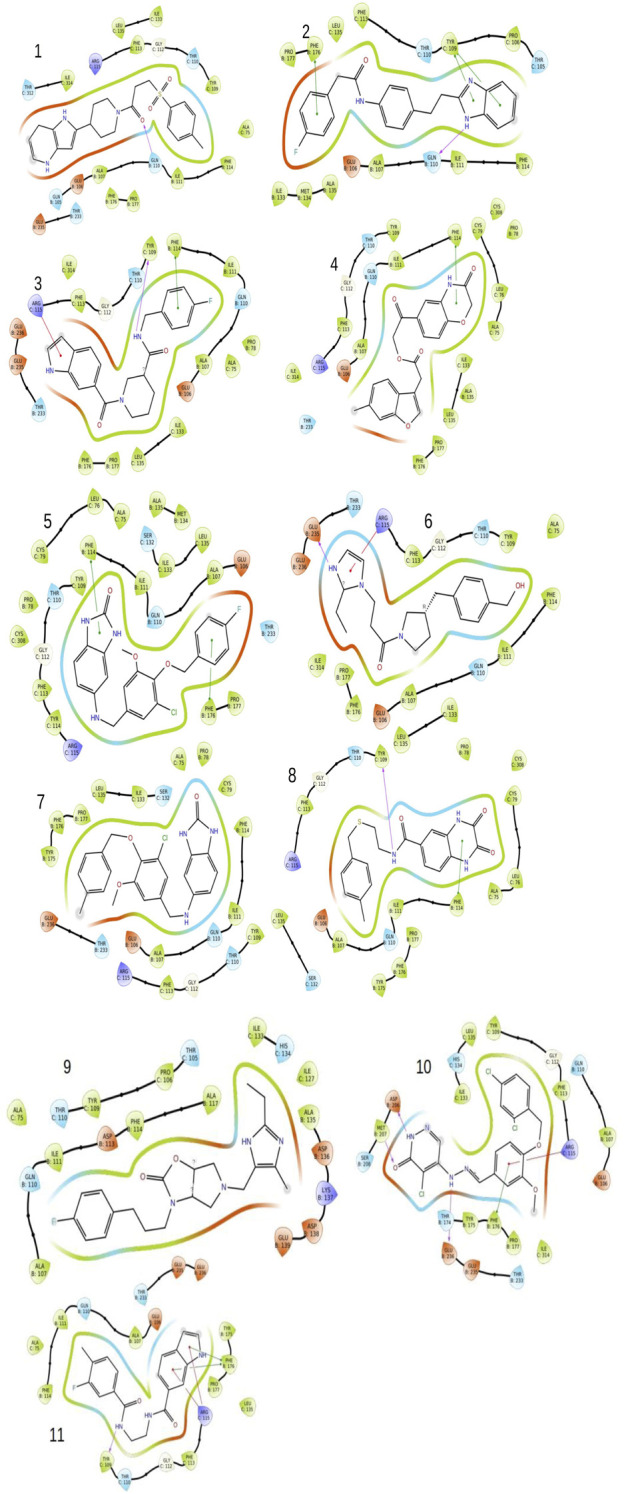
The image indicates the 2D plots of various shortlisted top inhibitor molecules after docking based evaluation.

### 3.5 Molecular dynamics simulation

**FIGURE 4:**
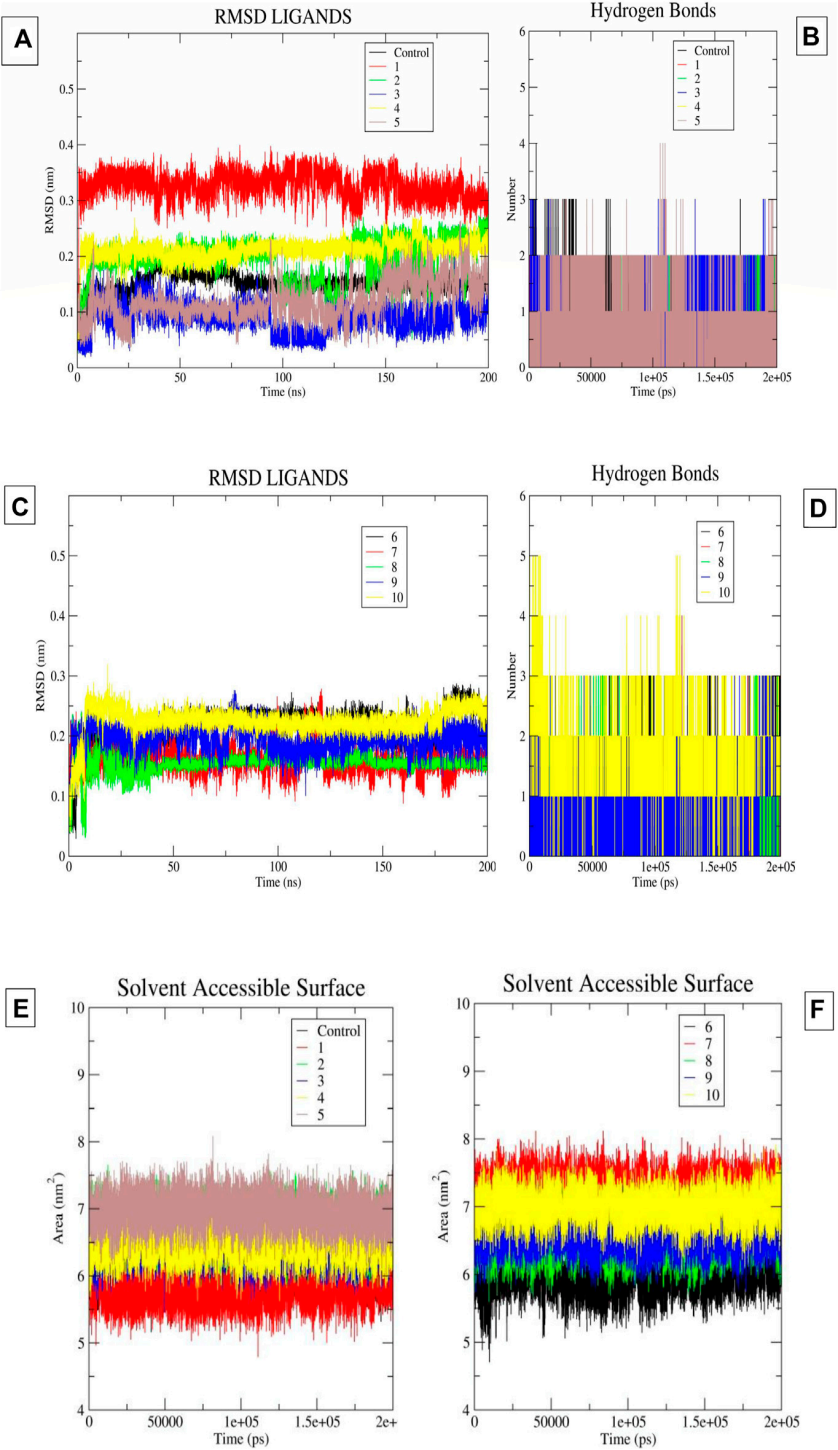
**(A–F)** The image 5A and 5B indicates the ligand RMSD and Hydrogen Bonds of compounds 1-5 and control molecule ifenprodil. Image 5C and 5D indicates the ligand RMSD and Hydrogen Bonds of compounds 6-10. Image 5E-5F indicates the SASA plots of ligand 1-10 and ifenprodil (control).

In the present study, we performed a 200 ns molecular dynamics simulation for each hit ligand. The corresponding interaction patterns were considered after the completion of the molecular dynamics simulation run. The RMSD of the ligand was calculated along with hydrogen bonding plots and solvent accessible surface areas (SASA) for each system using the trajectory file (Figures 4A–F). We carefully studied the trajectory of ifenprodil bound complex. We observed that compared to other fragment the benzyl fragment was showing slightly more fluctuation, but these deviations were below 3 Å. It was found that 7 ligands provided stable RMSD plots with respect to protein. The ligands, namely, 2, 3, 4, 6, 8, 9, 10, displayed highly stable poses with little deviation in the binding pocket of the protein. Most fluctuations were below 3. While the ligands, namely, 1, 5, 7, indicated an unstable RMSD with a constant deviations. Although for ligand 5 these deviations were observed to be within an acceptable range of less than 3. Observing the hydrogen bonding diagrams, it was found that the compound, namely, 2, 3, 5, 8, 9, 10, was constantly forming hydrogen bonds. The highest number of hydrogen bonds was observed in compound 10, followed by 9, 8, 5, and 3. For compounds 1 and 7, the observed hydrogen bonds were minimal, indicating their poor pose stability during MD simulation. The SASA plots showed that the SASA values for most of the compounds were comparable to those of the control molecule ifenprodil, except for compound 7, which was slightly on the high end. By carefully analyzing the RMSD diagrams, hydrogen bond diagrams and SASA values, it can be concluded that 5 molecules, namely, compounds 3, 5, 8, 9, and 10, are very stable due to hydrogen bonding interactions as well as especially hydrophobic interactions in the binding pocket with the residues, namely, Phe114 and Phe176. It was observed above mentioned 5 molecules mostly had Pi-Pi stacking interactions with the Phe114, Phe176 of chain B, while another interaction being hydrogen bond formation with Glu236 of chain B. Another key interaction was cation-Pi interaction with Arg115 of chain A. These interactions were mostly observed by the groups, namely, benimidazole, pyrrolo-pyridine and the benzoxazin-3-one group in the ligands.

### 3.6 Solvent based binding energy calculations

Using the 50 ns time frame of the trajectory of various protein-ligand complexes, we calculated the MM-PBSA score for the complexes. We observed that the molecules, namely, 3, 4, 5, 8, 9, 10 showed comparatively higher solvent-based binding affinities than the ifenprodil molecule. The observed affinity was −30.7786 kcal/mol for the control molecule. Notably, molecule 3 had the highest binding affinity −42.4383 kcal/mol. Molecules 9 and 10 had a very close binding affinity of −40.7174 kcal/mol and −40.8428 kcal/mol. From this it can be concluded that, namely, 3 to 10 all had relatively higher binding affinities than the control molecule ifenprodil. Binding affinities are summarized in Table 2.

## 4 Conclusion

In the current research, we used computational studies to find brand new NMDAR inhibitors. Using pharmacophore-based screening, we narrowed the search range to 11.32% by shortlisting molecules whose pharmacophore properties matched those of previously reported inhibitors. We were able to achieve very good precision and recall values for our models compared to the previously published pharmacological models, which shows that these models have a high chance of being used for shortlisting inhibitors. These final hits were further analyzed by docking-based studies to assess their binding affinity to the NMDAR. To our knowledge, the integration of these two methods for virtual screening of large databases against the NMDAR protein has not been previously reported. Finally, we shortlisted 10 molecules with a higher binding affinity than the control molecule Ifenprodil. In addition, we identified 5 molecules with stable RMSD plots, more hydrogen bonding, and higher binding affinities in a solvation-based scoring scheme than ifenprodil. The shortlisted molecules can be evaluated for their antiproliferative effects on NMDARs. By analyzing the interaction pattern of the selected top molecules in the extended MD run, they can be further optimized to increase potency. Furthermore, through MD studies we were able to identify 3 groups, namely, the benimidazole, pyrrolo-pyridine and the benzoxazin-3-one group, which turned out to be highly stable within the binding pocket of NMDAR. Also, it was observed that compounds which were showing higher temporal stability were mostly involved in Pi-Pi stacking interactions with the Phe114, Phe176 of chain B along with hydrogen bond interaction with Glu236 of chain B. Other crucial interaction we observed was cation-Pi interaction of ligands with Arg115 of chain A. The above mentioned groups as well as molecular interaction features could be further explored to develop new inhibitors with therapeutic potential against NMDARs. In addition, recent work can be used as a benchmark for the application of computational biology in the treatment of AD.

## Data Availability

The original contributions presented in the study are included in the article/Supplementary Material, further inquiries can be directed to the corresponding author.
