# Supplementary material for: Targeting NMDA receptor in Alzheimer’s disease: identifying novel inhibitors using computational approaches
Source: Front Pharmacol. 2023 Jun 21;14:1208968. doi: 10.3389/fphar.2023.1208968 (PMC10319995; doi:10.3389/fphar.2023.1208968)
Supplement: Supplementary file 1 [file DataSheet1.docx]

Supplementary Data:

**
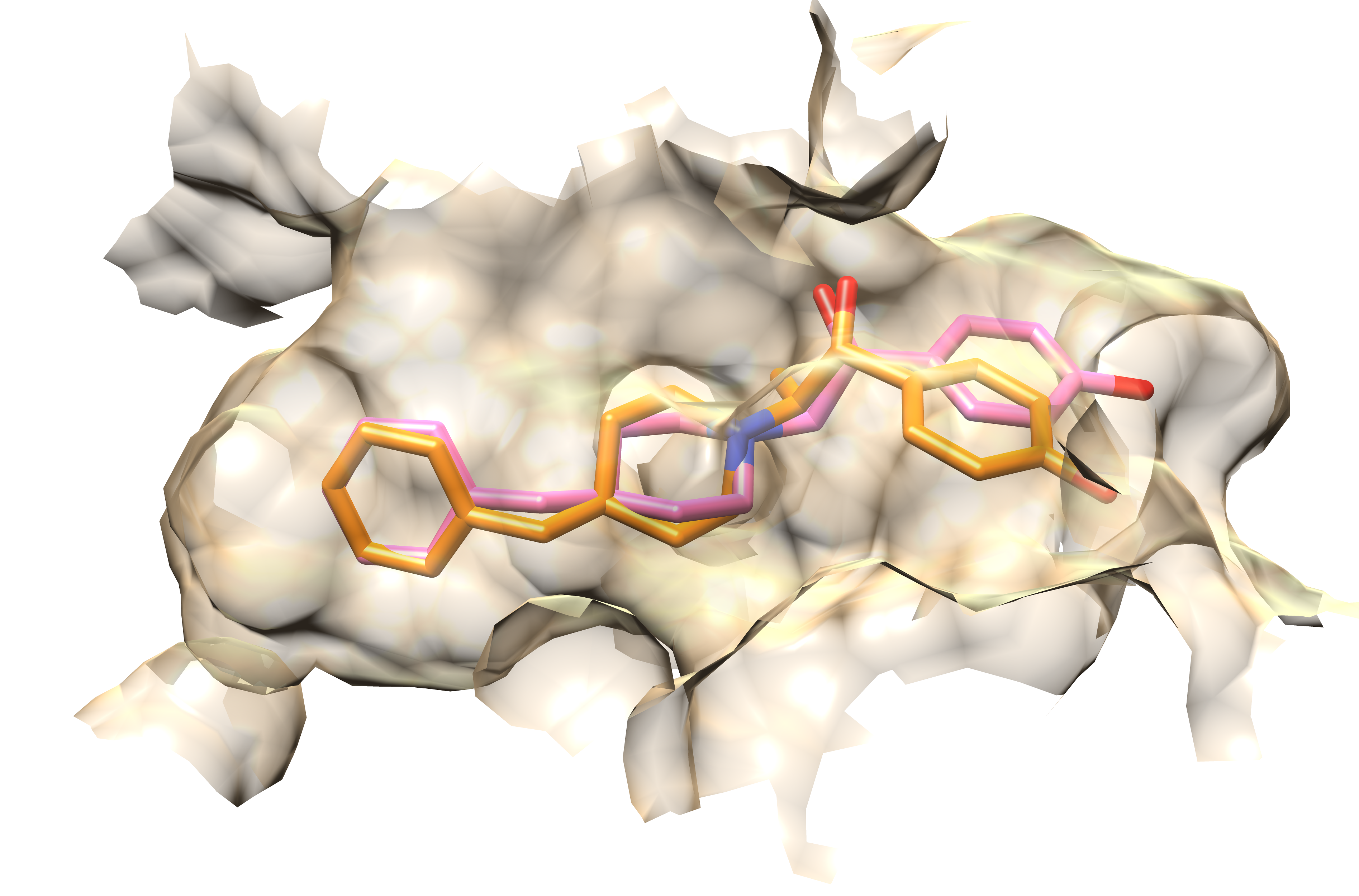
**

**Figure S1:** The crystal bound (hot pink) and redocked (orange) orientation of ifenprodil within the active site of NMDAR.
